# Peri-Implantitis: Application of a Protocol for the Regeneration of Deep Osseous Defects. A Retrospective Case Series

**DOI:** 10.3390/ijerph182312658

**Published:** 2021-12-01

**Authors:** Simone Verardi, Nicola Alberto Valente

**Affiliations:** 1 Department of Periodontics, University of Washington, Seattle, WA 98195, USA; simover@yahoo.it; 2 Department of Periodontology, School of Dental Medicine, University of Cagliari, 09124 Cagliari, Italy; 3 Department of Periodontics and Endodontics, State University of New York at Buffalo, New York, NY 14214, USA

**Keywords:** peri-implantitis, tetracycline, glycine, xenograft

## Abstract

Background and aims: Peri-implantitis is a complex pathology, both in its diagnosis and in the identification of etiological causes. Although we have been studying more and more over the years to try to answer the many questions that remain regarding everything that circulates around this disease which affects implants, nothing has yet been taken as an official consensus regarding its surgical treatment. There are still many proposed protocols, each of which has been shown to have comforting results and promising prospects, but no total predictability. The aim of this case series is to assess the clinical outcomes of a mixed protocol for the regeneration of deep osseous defects. Materials and methods: The data and clinical records of 23 patients, with 29 implants affected by peri-implantitis treated surgically in private practice, were analyzed retrospectively. The method used for the surgical treatment was a mixed protocol of mechanical–chemical decontamination and bone regeneration with bovine xenograft. Results: All patients were followed for at least 2 years, averaging 28.9 months (a range of 24–38 months) with a reduction in the probing depth (PD) at one year from the initial 8.14 ± 1.156 mm to 3.72 ± 0.649 mm, and to 4.14 ± 1.093 mm at the final assessment. The differences between assessment time points were always statistically significant. The data regarding bleeding on probing (BoP) and suppuration also showed a statistically significant reduction at the final time point compared to the baseline. Only one patient, at 24 months, still showed BoP, suppuration, and a PD deeper than 5 mm, indicating a recurrence of the disease compared to the initial improvement of the PD (5 mm) at one year. Conclusions: In conclusion, within the limits of this retrospective analysis, it can be affirmed that this combined mechanical–chemical and regenerative decontamination therapy is effective in the treatment of peri-implantitis.

## 1. Introduction

Peri-implantitis disease was accurately defined as “A pathological condition occurring in tissues around dental implants, characterized by inflammation in the peri-implant connective tissue and progressive loss of supporting bone” [1]. The term was initially used in the 1960s in French literature as an osteolysis affecting the area that surrounded an implant. This was before titanium root-shaped devices became popular for the treatment of edentulous areas and thus was probably related to metals used at that time for dental implants with different pathogeneses [2].

Even if the etiology of this disease is not totally clear and many factors may be involved with the inflammation (implant surface, residual cement, periodontitis, previous local inflammation, lack of keratinized mucosa, etc.), the role of pathogenic bacteria is well documented [3,4,5].

The pathological process always starts with peri-implant mucositis, which is an inflammation that only affects the soft tissue around the implant. It is conventionally treated in a conservative way, simply by improving home and professional oral hygiene and, in some cases adding local antibacterials [1,6]. Its incidence is quite high [7], but this pathological condition is usually reversible [8,9].

The situation is more complicated to treat when bone is also affected, such as in peri-implantitis. Rapid bone destruction is also due to the particular immune response that is seen in this inflammatory process [10,11]. It is hard to correctly evaluate the prevalence of peri-implantitis, since the many studies conducted over the years used different diagnostic criteria, reporting a range between 0 and 39.7% [12].

Increased Probing Depth (PD), presence of Bleeding on Probing (BOP), and/or pus or swelling are the usual clinical signs of peri-implant inflammation. Radiographically, a bone lesion showing circumferential involvement is the most typical sign. A final consensus was recently reached on the diagnostic criteria (and thresholds when previous examinations or radiographs are not available) using the above-mentioned parameters to be considered for the diagnosis of such a disease [13]. However, an actual classification of the disease considering possible etiologies and degrees of manifestations is still lacking.

Many treatment options have been proposed during the years in areas affected by peri-implantitis [14]. It is widely accepted that mild cases should be treated non-surgically; this approach is also recommended as the initial phase for severe cases. It usually consists of non-surgical debridement with specific implant curettes, sprayed glycine, and local antibiotics delivery [15]. Several authors suggest surgical treatment for cases in which limited improvement is evident after the non-surgical treatment. Many surgical approaches have been proposed, including resective or regenerative procedures, usually in combination with decontamination processes [16,17,18,19].

Here, we report the 3-year result of a protocol used on 23 patients and 29 implants with a minimum follow-up of 24 months. The aim of this case series is to assess the clinical outcomes of a mixed protocol for the regeneration of deep osseous defects.

## 2. Materials and Methods

This study was conducted in accordance with the Declaration of Helsinki, as revised in 2013.

**Figure 1:**
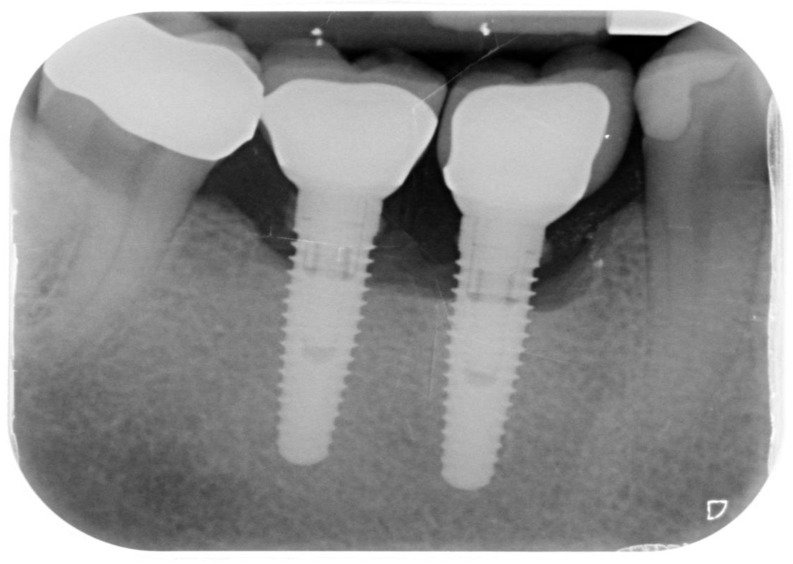
Periapical radiograph of implants affected by peri-implantitis in the lower right quadrant.

Records from 23 patients who had received treatment for peri-implantitis on one or more implants were retrospectively examined in the period between 24 and 38 months after their surgical treatment. A total of 29 implants were examined retrospectively. Patients were included if their records clearly showed that they had peri-implant bone defects on one or more implants treated with the method described in this retrospective study. The records were excluded in case of missing or incomplete pre-treatment, follow-up or final data, absent radiographs (Figure 1), or surgical/regenerative protocols different from that described.

**Figure 2:**
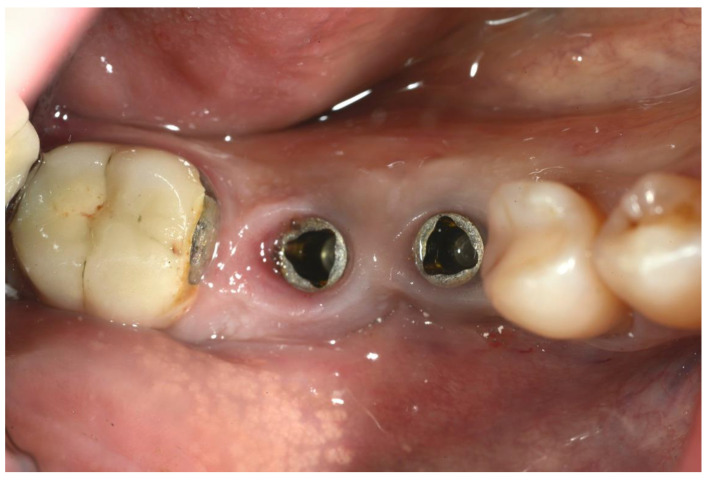
Preoperative image of the surgical area.

All patients had previously been treated non-surgically, and only those patients with Probing Depth > 6 mm and lack of implant mobility had been considered for surgical treatment. In order to be treated with regenerative surgical procedures, patients had to be non-smokers or smoke fewer than 10 cigarettes per day [20]. All the patients received a professional hygiene session 2–3 days before the surgical treatment; this was performed to reduce the intraoral bacterial load. The proposed treatment consisted of a combination of surgical and antimicrobial therapy. All patients received antibiotic prophylaxis with 2 g amoxicillin 1 h prior to the surgery. Before the procedure, all patients had a 1 min mouth rinse with 0.2% chlorhexidine. Articaine 4% with 1:100,000 adrenaline was used for the local anesthesia by infiltration; mandibular block was also used in the mandible (Figure 2).

**Figure 3:**
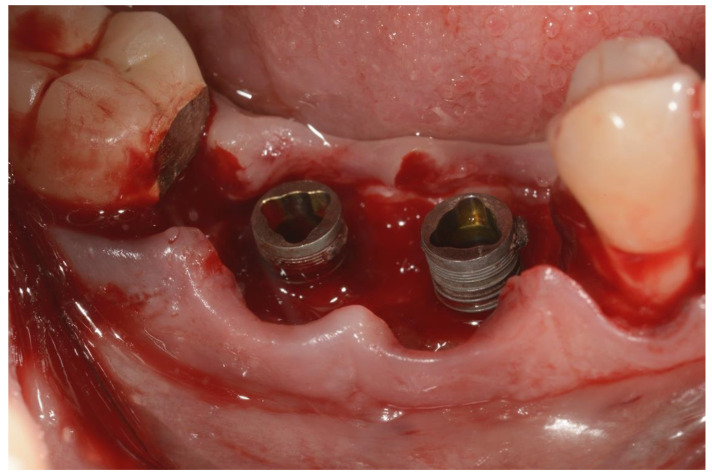
Surgical area after incision and flap elevation.

An incision was made on the crest and vertical releasing incisions on the mesial and distal buccal sides. After elevating a muco-periosteal flap, the granulation tissue around the affected implant was then removed using surgical curettes (Figure 3).

**Figure 4:**
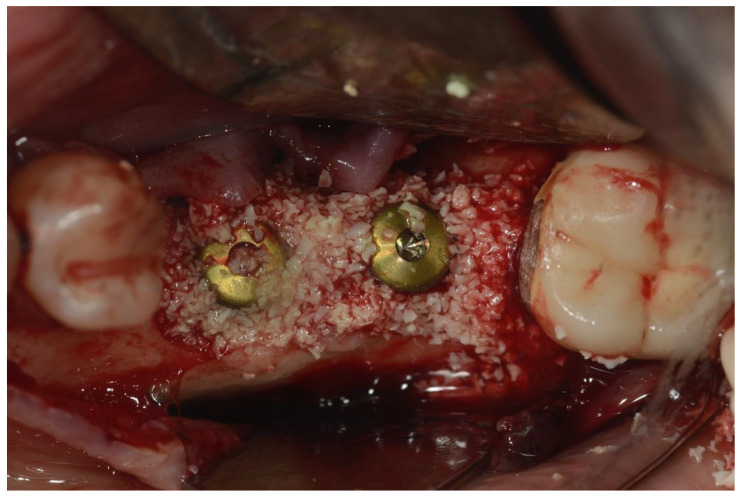
Placement of bovine particulate graft material.

Once the implant and the osseous defects were exposed and accessible, a Teflon insert mounted on a Piezoelectric surgical device (Piezosurgery Mectron. Carasco, Italy) was used to remove debris and calculus on the implant surface. The tip of the insert was used to reach all the narrow parts, including the space between threads, without scratching the implant surface. This part of the procedure was continued until the elimination of all the macroscopic hard debris such as calculus, cement, and necrotic bone or bone substitutes particles [3]. Chemical decontamination, using a 35% phosphoric acid gel, was then performed [21]. Attention was paid to make sure that the gel was only applied on the metal surface, avoiding bone and soft tissues. The gel was left in place for about 2 min, then suctioned, and the surface was cleaned by copious irrigation with saline solution (B. Braun, Melsungen AG, Germany) for 60 s. Glycine powder with 25 μm particle size (Perio. EMS. Nyon, Switzerland) was then sprayed on the implant surface for about 30 s using the dedicated handpiece (Airflow, EMS. Nyon, Switzerland) [22]. A tetracycline hydrochloride powder was then mixed with saline solution (B. Braun, Melsugen AG, Germany) and applied on the surgical site for about 2 min [23]. Another cycle of copious irrigation with saline solution (B. Braun, Melsugen AG, Germany) for 1 min followed the previous step. After making sure that no soft tissue or hard tissue debris was still present, a bovine xenograft (Bios, Geistlich) was placed in the osseous defects and then covered with a resorbable collagen membrane (Figure 4).

**Figure 5:**
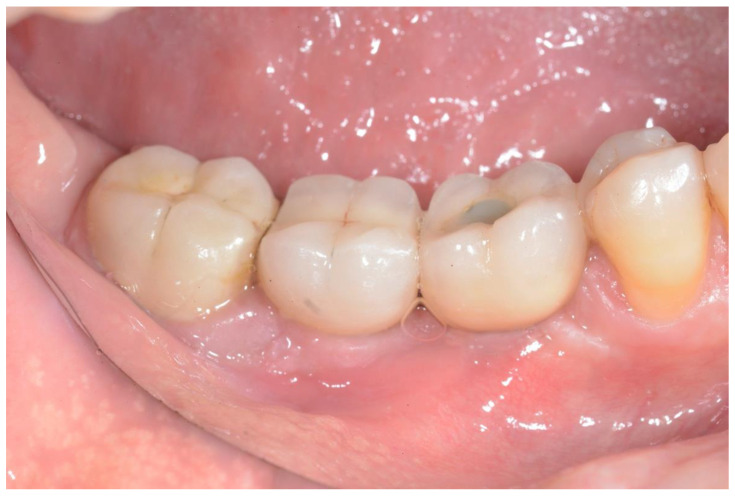
Clinical appearance at the 38-month follow-up.

The flap was then released by sharp dissection on the buccal side in order to have a passive adaptation, and it was sutured using monofilament sutures. A combination of vertical mattress sutures and single interrupted sutures allowed flap stability and closure. Patients were told to place an ice pack at 10 min intervals and not to brush on the treated area until 2 days after suture removals. The post-op prescription included: Amoxicillin + Clavulanic Acid 1 g tabs b.i.d. for 7 days, Metronidazole 250 mg tabs t.i.d for 7 days, Ibuprofen 600 mg tabs b.i.d. for 3 days, and chlorexidine mouth rinse 0.2% b.i.d. for 15 days. Vitamin B complex and vitamin D were also prescribed: 1 pill a day for 20 days for the first supplement, and 25,000 IU vials per os every 15 days for the Vitamin D. Patients were seen 7 days after the surgical treatment and then again 14 days after the surgery for suture removal. Follow-up exams were then scheduled every 2 months for the first 6 months and then every 6 months, unless required (Figure 5).

**Figure 6:**
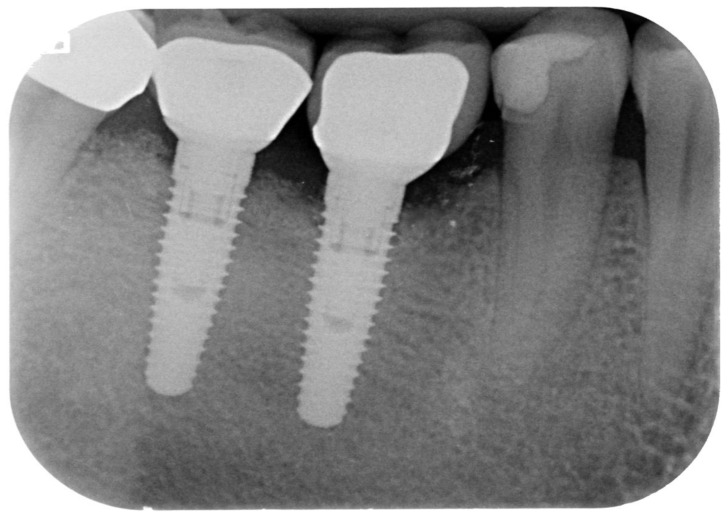
Follow-up radiograph (38 months).

**Figure 7:**
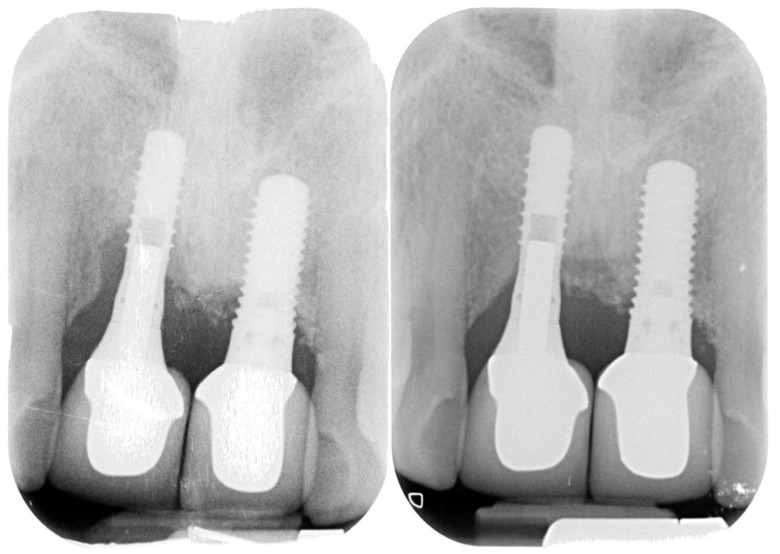
Peri-implantitis affecting upper central incisors and clinical resolution at the 35-month recall.

Periapical radiographs were taken at the 6-month and 12-month control visits and then yearly (Figure 6 and Figure 7).

All patients were put on a strict supportive therapy protocol with periodontal recalls every 3 months.

### Statistical Analysis

Descriptive statistics were calculated, including means, SDs, medians, and confidence intervals. PD measurements at baseline and one year at final follow-up were analyzed using the one-way repeated measures ANOVA test, while the statistical analysis of the BoP and suppuration values at baseline and final evaluation was made using McNemar’s test. The level of significance was established at 5% (*p* = 0.05), and the analysis was carried out using statistical software (SPSS version 26, IBM).

## 3. Results

The group population and baseline values are summarized in Table 1.

The study population consisted of 11 males and 12 females with a mean age of 53.8 when they received the surgical therapy. Nine patients were smokers at the time of the surgical therapy, and 11 had a history of periodontitis. At the baseline, the mean Probing Depth was 8.14 ± 1.156 mm; Bleeding on Probing (BOP) was present on 28 out of 29 treated areas; pus was instead present on 15 out of 29 sites. At the time of surgical treatment, all patients were ASA 1 and 2. They had received detailed written and oral information about the possible benefits of the treatment, as well as the risks, and they had all given written informed consent.

All patients had a confirmed diagnosis of peri-implantitis based on the presence of Bleeding on Probing (BOP) and/or pus, localized edema, erythematous peri-implant mucosa, and radiographic signs of bone loss.

Twenty-three patients who had been surgically treated for peri-implantitis affecting 29 implants and followed for a minimum of 24 months were included in this retrospective analysis; the mean follow-up time was 28.9 months (a range of 24–38 months). All outcomes are summarized in Table 2.

A repeated-measures ANOVA, with a Greenhouse–Geisser correction applied for the non-sphericity of the data revealed by the Mauchly’s Test (*p* = 0.025), determined that mean PD values differed significantly between time points (F(1.614, 45.186) = 295.201, *p* < 0.0005). Post hoc tests using the Bonferroni correction revealed that the peri-implantitis treatment elicited a significant reduction in PD from the baseline to the one-year follow-up (8.14 ± 1.156 mm vs. 3.72 ± 0.649 mm, respectively), which was significant (*p* < 0.0005). However, the final PD showed a mean value of 4.14 ± 1.093 mm, which was statistically significantly different from the baseline (*p* < 0.0005) and from the one-year follow-up, although only slightly (*p* = 0.047).

At the time of final examinations, BOP was present on 7 out of 29 sites; pus was present only on one surgical site. An exact McNemar’s test determined that there was a statistically significant difference in the proportion of implants with BoP and pus pre- and post-intervention (*p* < 0.0005 and *p* = 0.001, respectively).

## 4. Discussion

In this retrospective analysis of data from clinical records, 23 patients were treated with a mixed protocol based on mechanical debridement, chemical/pharmacological decontamination, and bone regeneration. The mean PD went from the initial 8.1 mm to 4.1 mm. One implant (#18) had a negative outcome because the clinical conditions worsened with time and returned to the initial conditions. All the other implants had improvement compared to the baseline.

The results, as shown by the PD reduction already at 12 months and also at the final follow-up, are satisfactory in terms of containment of the disease progression. Data relating to BoP and pus confirm the reduction of inflammation and infection. In six patients, there was some relapse of PD, but only in one of these was the probing depth severe (8 mm), with the simultaneous presence of bleeding and pus. In the remaining cases, the relapse was minimal (1 mm), with PD all between 4 mm and 5 mm and residual BOP in only one case. Therefore, only one case could be considered a failure of the decontaminative/regenerative therapy, although the implant was still in place. The causes of failure can be many, from the patient’s non-compliance to the incomplete decontamination of the surface, whose total sterility is neither predictable nor verifiable [24]. Additionally, the use of a grafting material introduces an additional variable that can determine a greater probability of failure if an infection occurs during healing [25].

The initial degranulation of the defects was performed to fully expose the defect resulting from bone resorption; this first and essential phase of the treatment was performed with a surgical curette first to perform a gross removal. A Teflon piezoelectric tip was then used to perform a more accurate removal on the implant surface without damaging it. The non-aggressiveness of the coated ultrasonic tips is demonstrated both in vitro and in vivo. A study by Rühling et al. [26] showed how the titanium surfaces treated with Teflon-coated ultrasonic tips were, at the SEM analysis, similar to the control surfaces and also that these inserts guaranteed less overheating. More recently, in a multicenter study on 89 patients, Blasi et al. [27] demonstrated that plastic-coated tips provided statistically comparable, if not clinically superior, results to other commonly used mechanical debridement methods.

After ultrasonic degranulation, we applied phosphoric acid only on the surface of the implants, making sure that the substance did not come into contact with hard and soft tissues. This was performed in order to further eliminate as much calculus residue as possible remaining between the micro-threads of the implants, and at the same time, carry out initial decontamination. In a very recent RCT by Hentenaar et al. [21] on 28 patients with implants affected by peri-implantitis, the application of 35% phosphoric acid was superior to mechanical debridement with rinsing of saline solution (B. Braun, Melsugen AG, Germany). Furthermore, an in vitro study showed that implants treated with 37% phosphoric acid incubated with human blood mononuclear cells for 24 h showed higher cell viability rates and an increase in the levels of IL-2, IL-4, IL-6, IL-10, and TNF-α, thus suggesting that it can modulate the immune response, thereby improving biofunctional processes and promoting the dental implants’ success [28].

To ensure the removal of any residual phosphoric acid, abundant irrigation with saline solution (B. Braun, Melsugen AG, Germany) was applied for 60 s, followed by a spray with 25 μm glycine powder with a dedicated handpiece. Air polishing with glycine powder is effective in implant cleaning without modifying chemical and elemental compositions and the biocompatibility of implant surfaces, as demonstrated in vitro by Bennani et al. [22]. Air polishing is also equally clinically effective as implantoplasty, and less harmful on surfaces than scaling with manual curettes or the sonic scaler [29,30].

Finally, to ensure further disinfection of the implant surface but also of the bone defect that surrounds it, tetracycline powder diluted in saline solution (B. Braun, Melsugen AG, Germany) was applied to the entire defect. The dilution of tetracycline allows one to avoid the potential decrease in cell viability that this drug can give at high concentrations, thus favoring not only its antimicrobial activities, but also its effect on osteoblastic activity by inhibiting matrix metalloproteases (MMPs) [31]. Tetracycline has often proved to be an effective aid in the decontamination of surfaces in peri-implant defects, in vitro and in vivo, precisely by virtue of its simultaneous antibiotic and inhibitory action of osteoclastic differentiation [23,32,33].

The peri-implant bone defects were then grafted with bovine xenograft and covered with a collagen membrane. This type of graft has proven effective in several clinical studies when used for peri-implant bone defects, with rather long follow-ups, especially when adequate maintenance therapy is provided and patients are compliant [34,35,36].

Finally, the patients were prescribed post-surgical therapy based on antibiotics and vitamin supplements. Antibiotic prophylaxis, especially that based on a mix of amoxicillin and metronidazole, is widely used by clinicians for the treatment of peri-implantitis with excellent results [37], and in a retrospective clinical study, it has been shown to have superior results in terms of BoP and mucosal recession compared to the group of patients who did not receive systemic antibiotics [38]. Moreover, in a randomized clinical trial, the use of systemic metronidazole elicited excellent results in clinical, radiographic, and microbiological parameters in the non-surgical treatment of peri-implantitis [39]. The vitamin supplement has been shown, both in vitro and in clinical studies, to be a very useful support in the healing of surgical wounds [40]; moreover, a retrospective clinical study has shown how the vitamin supplement has proved useful in countering antibiotic-associated pseudomembranous colitis [41]. Therefore, the use of vitamin supplements is even more justified in the light of the prescribed antibiotic prophylaxis.

Other variables had to be considered when treating patients with the suggested protocol; heavy smokers were not considered good candidates and thus were not treated with regenerative procedures. The same limitation was adopted for some patients who reported mild to severe systemic diseases (ASA class II and higher). No specific parafunctional habits were diagnosed; however, most of the patients were already wearing a night guard for protection of the prosthetic appliances. No difference was noted between patients wearing or not wearing the night guards. The strict patient selection that the authors used might have favored the positive results that were observed.

There is no evidence in the literature to support the superiority of one treatment for peri-implantitis over another [42]; however, in our retrospective analysis of 23 cases and 29 implants, the authors have shown how the combination of different strategies, all supported by scientific evidence, lead to more than acceptable and maintainable results in the medium term. We can say that the combination of several chemical–mechanical decontamination methods has probably contributed to ensuring better results in maintaining clinical parameters within healthy ranges, and probably favoring the positive outcomes of regenerative therapy, all without leading to detrimental effects.

## 5. Conclusions

Within the limits of this clinical report, documented through the cases reported in this retrospective analysis, it can be concluded that a combined chemical–mechanical and regenerative decontamination therapy is effective in the treatment of peri-implantitis. Further investigation, including a control group and/or histologic findings, may be needed in order to confirm the clinical results that were reported.

## Figures and Tables

**Table 1:** Baseline values.

Patients	Implants	Sex	Mean Age	Smokers (n.)	History of Periodontitis (n)	BoP	Pus	Mean PD
23	29	11/12	53.78	9	11	28	15	8.14 ± 1.156 mm

**Table 2:** Outcome values.

	Baseline			One Year			Final			
	Mean + SD (mm)	Median + MAD (mm)	95% CI	Mean + SD (mm)	Median + MAD (mm)	95% CI	Mean + SD (mm)	Median + MAD (mm)	95% CI	
PD	8.14 ± 1.156	8 ± 1	7.7, 8.6	3.72 ± 0.649	4 ± 4	3.5, 4	4.14 ± 1.093	4 ± 4	3.7, 4.5	*p* < 0.0005
	n. of sites						n. of sites			
BoP	28						7			*p* < 0.0005
Pus	15						1			*p* = 0.001

## Data Availability

Data supporting the reported results are stored in a locked archive in the institution to which the author NAV is affiliated.
